# Labs in the time of COVID: an early-career scientist's view

**DOI:** 10.1242/dmm.046151

**Published:** 2020-06-23

**Authors:** Ling-shiang Chuang

**Affiliations:** 1 Department of Genetics and Genomic Sciences, Icahn School of Medicine at Mount Sinai, New York, NY 10029, USA; 2 The Charles Bronfman Institute for Personalized Medicine, Icahn School of Medicine at Mount Sinai, New York, NY 10029, USA

## Abstract

The outbreak of COVID-19 has stalled both the basic, clinical and non-COVID medical research. The scientific community has shown extraordinary flexibility and resilience in responding to the pandemic. However, funding restructuring, risk of infection, cancelation of scientific conferences and delayed experiments have already proven detrimental to the career opportunities of early-career scientists. Moreover, school closures and a lack of systematic support for childcare have been additional challenges for early- and mid-career researchers who have young children. This Editorial describes an early-career researcher's experience and highlights how after efficiently contributing to ‘flattening the curve’ of COVID-19 infections, the research community has an opportunity for growth and re-structuring.

## Abstract (precis)

**Summary:** The COVID-19 outbreak has created a global health crisis that is profoundly affecting early-career scientists. This Editorial describes an early-career researcher's experience and the professional and personal challenges during the pandemic.

Back in February, everyone in New York City felt that it was inevitable that the city would become a part of the COVID-19 pandemic. This city, and its research institutions, are known for their highly competitive culture and financial pressures. With a dense population and convenient public transportation, it was impossible for residents to adequately ‘respond’ by merely attempting to change their routine. The first COVID-19 case in NYC was treated in the emergency room at Mount Sinai Hospital, the same institute where our laboratory is based, and the numbers quickly escalated from there. This made Mount Sinai one of the clinical epicenters of the outbreak. On 20 March, all the research labs at Mount Sinai, except those conducting COVID-19 studies, were shut down. A week later, our hospital admitted more than 2200 COVID-19 patients daily, which was the peak of the outbreak.

It was a difficult time for bench researchers, as the ‘stay-at-home’ order brought about many uncertainties. Our lab tried to collect as much wet-lab data as possible when we realized the institutional shutdown was inevitable. Multiple runs of single-cell RNA sequencing and thousands of zebrafish images were collected a week before the closing. So, we planned on using the ‘stay-at-home’ time for data analysis and manuscript submission. During the shutdown, only essential personnel were allowed in the animal facilities to perform basic husbandry. Mount Sinai shifted all its resources to COVID-19 research, so more data and new technology could support first-line clinicians in their fight against COVID-19. Although we are not an infectious disease lab, we became actively involved in COVID-19 research by providing our previously collected patient serum samples for NIH projects. We also contributed a COVID-19-positive serum sample for antibody assay development at Georgia State University.

I am an instructor at Mount Sinai, meaning I am still an early-career researcher, and COVID-19 has negatively affected my career development, my research work and our hospital. Firstly, one of my signature publications, which was published last year in Disease Models & Mechanisms (Chuang et al., 2019), focused on the development of a novel inflammatory bowel disease (IBD) zebrafish model to mimic the acute and chronic inflammation in a live fish gut. Back in March this year, I was invited by the Crohn's and Colitis Foundation, an important funder in our field, to Gilead Pharmaceuticals for in-depth discussions and future collaborations on using our novel zebrafish model for IBD drug screening. It was supposed to be a dream-come-true moment to contribute to drug discovery. But, just like all the scientific conferences, this meeting was canceled three days before I was due to fly to San Francisco. It was a big disappointment. I felt like I had been running a marathon but was unable to cross the finish line.

Secondly, since we were regularly collecting human intestinal tissue samples for our research, the post-COVID-19 regulations on tissue-procurement processes, such as biopsies or resections, further complicate our day-to-day functioning. Specifically, even in recovering COVID-19 patients that tested negative, SARS-CoV-2 RNA was detected in stools or gut tissues for more than 30 days. This prolonged viral presence is making both clinical endoscopy and GI research challenging in the post-COVID era. We will all need to adjust to this new normal once the labs fully reopen.

Thirdly, all non-COVID related medical and basic research was struck hard. Hospitals are losing revenue due to canceled surgeries and treatment procedures, disease-focused foundations withdrew their summer calls for career development and other awards, and most government grants are moving toward funding COVID-19 research instead. These conditions have further narrowed the already scarce funding pool available to early-career scientists. Moreover, many elite journals are prioritizing providing up-to-date information for COVID-19 and have set aside other medical research. I have a pilot grant, which is due in July this year. With the current delay, I need to negotiate with our grants officer to find a way to resolve the problem. Luckily, it will only take a week to scale our zebrafish lines back to our routine to resume experimental work, so my delay will be fairly short. My colleagues working on mouse models will need months or up to a year to get back to their experiments.

However, not all is bad. With web-based video conferencing tools and secure online data sharing software, our lab has managed to keep our scholastic activities such as journal club and lab meetings running weekly. The departmental and institutional work-in-progress presentations also continued without interruption. Mount Sinai is now in Phase I of its reopening plan, so we are currently open at 25% capacity in the lab. This means one person per research bay to comply with social distancing guidelines. Researchers have to wear a mask the whole time. To help the group stay fully compliant with all the new COVID-19-related laboratory regulations, we developed a shared Google sheet.

With two young daughters at home, it is tough to maintain the same work efficiency as in the lab. My wife, Nai-Yun, who is a post-doctoral fellow, and I break the day into sections according to our kids’ online school schedule. With a clear to-do list every day, we can focus on our tasks when the kids are occupied with their school work or assignments. However, there are more challenges to come: with schools staying closed and summer camps canceled, we desperately need childcare support for the next phase of reopening and for when our research facilities return to full capacity. We are lucky to have a kind nanny who is willing to take care of our kids during our work time, even though she knows that helping us increases her risk of catching COVID-19. However, the extra financial burden of private childcare will keep us on an even tighter budget for the rest of the year.

My PI, Judy Cho, is exceptionally supportive. At the beginning of the pandemic, when there was not much information on how to continue with research, she scheduled multiple teleconferences with specialized COVID-19 GI clinicians, so we could get first-hand information on how to protect our lab members adequately. We frequently discussed new findings in our lab meetings, so every member had up-to-date information. We shut the lab down early, and now we are carefully following the reopening plan with a weekly supply of personal protective equipment from our department and the hospital.

As I mentioned in the beginning, uncertainties remain. Based on the current epidemiological model, COVID-19 is not going away any time soon. There is a high possibility of a second wave of infections, and we still have a lot to learn from this highly contagious virus. However, with full implementation of personal protective equipment, social distancing policies, and regular virus and antibody testing, we now can push research forward without risk or fear. The research community has shown extraordinary flexibility and resilience in responding to the pandemic. However, this crisis has also exposed several systemic issues, like funding and provisions for early- and middle-career researchers, that we now have the opportunity to re-think.

See also the First Person interview accompanying this article.

## References

[DMM046151C1] Chuang, L., Morrison J., Hsu N., Labrias P. R., Nayar S., Chen E., Villaverde N., Facey J. A., Boschetti G., Giri M., Castillo-Martin M., Thin T. H., Sharma Y., Chu J. and Cho J. H. (2019). Zebrafish modeling of intestinal injury, bacterial exposures and medications defines epithelial in vivo responses relevant to human inflammatory bowel disease. *Dis. Model. Mech.* 12, dmm037432 10.1242/dmm.037432. 31337664 PMC6737949

